# Multiple β-Lactam Resistance Gene-Carrying Plasmid Harbored by Klebsiella quasipneumoniae Isolated from Urban Sewage in Japan

**DOI:** 10.1128/mSphere.00391-19

**Published:** 2019-09-25

**Authors:** Yasunori Suzuki, Miki Ida, Hiroaki Kubota, Tsukasa Ariyoshi, Ko Murakami, Makiko Kobayashi, Rei Kato, Akihiko Hirai, Jun Suzuki, Kenji Sadamasu

**Affiliations:** a Department of Microbiology, Tokyo Metropolitan Institute of Public Health, Tokyo, Japan; Escola Paulista de Medicina/Universidade Federal de São Paulo

**Keywords:** carbapenemase-producing *Enterobacteriaceae*, conjugal transfer, metallo-β-lactamase, plasmid, whole-genome sequencing, *Enterobacteriaceae*, beta-lactamases, carbapenems, conjugation, genome analysis

## Abstract

The continuous emergence of carbapenemase-producing *Enterobacteriaceae* (CPE) presents a great public health challenge. Mitigation of CPE spread in the environment is crucial, particularly from a One Health perspective. Here we describe the isolation of CPE strain SNI47 from influent water of a sewage treatment plant in Japan. SNI47 was identified as Klebsiella quasipneumoniae subsp. *quasipneumoniae* by phylogenetic analysis and was resistant to β-lactams, including carbapenems. Of four plasmids detected from SNI47, the 185,311-bp IncA/C2 plasmid (pTMSNI47-1), which carried 10 drug resistance genes, including genes for four β-lactamases (*bla*_CTX-M-2_, *bla*_DHA-1_, *bla*_KHM-1_, and *bla*_OXA-10_), was transferred to Escherichia coli J53 via conjugation. The MICs of all tested β-lactams for the transconjugant were higher than for the recipient. We constructed recombinant plasmids, into which each β-lactamase gene was inserted, and used them to transform E. coli DH5α cells, demonstrating that KHM-1 enhanced carbapenem resistance. In addition, these β-lactamases were responsible for a wide-spectrum β-lactam resistance acquisition with mutual compensation. KHM-1, recognized as a rare type of metallo-β-lactamase, was detected in a transferable plasmid, from a sewage treatment plant, involved in horizontal gene transfer. The detection of such plasmids raises a health risk alarm for CPE dissemination.

**IMPORTANCE** In our investigation of urban wastewater in Japan, carbapenem-resistant Klebsiella quasipneumoniae subsp. *quasipneumoniae* was isolated that carried the pTMSNI47-1 plasmid, which carries four β-lactamase genes and has transferability among *Enterobacteriaceae*. pTMSNI47-1 was found to encode a rarely reported carbapenemase, KHM-1. Cooperative effects of β-lactamases encoded by pTMSNI47-1 appeared to have broad-spectrum resistance to β-lactams. The detection of the KHM-1 gene in urban wastewater suggests that such a rare antimicrobial resistance (AMR) gene can be pooled in the environment, potentially emerging as an AMR determinant in a pathogen. When the number of β-lactamase resistance genes is increased in one plasmid, the transfer of this plasmid can confer broad-spectrum resistance to β-lactams, even if the individual gene confers narrow-spectrum resistance. The present study adds important information about the potential risk of sewage treatment plants as reservoirs and environmental suppliers of AMR genes, contributing to the public health from a One Health perspective.

## Abstract (precis)

In our investigation of urban wastewater in Japan, carbapenem-resistant Klebsiella quasipneumoniae subsp. *quasipneumoniae* was isolated that carried the pTMSNI47-1 plasmid, which carries four β-lactamase genes and has transferability among *Enterobacteriaceae*. pTMSNI47-1 was found to encode a rarely reported carbapenemase, KHM-1. Cooperative effects of β-lactamases encoded by pTMSNI47-1 appeared to have broad-spectrum resistance to β-lactams. The detection of the KHM-1 gene in urban wastewater suggests that such a rare antimicrobial resistance (AMR) gene can be pooled in the environment, potentially emerging as an AMR determinant in a pathogen. When the number of β-lactamase resistance genes is increased in one plasmid, the transfer of this plasmid can confer broad-spectrum resistance to β-lactams, even if the individual gene confers narrow-spectrum resistance. The present study adds important information about the potential risk of sewage treatment plants as reservoirs and environmental suppliers of AMR genes, contributing to the public health from a One Health perspective.

## INTRODUCTION

Worldwide, infections caused by carbapenemase-producing *Enterobacteriaceae* (CPE) are of utmost interest in clinical settings because carbapenems are often antimicrobial agents of last resort (1, 2). Moreover, it is necessary to mitigate CPE spread in the environment, particularly from a One Health perspective. Nevertheless, CPE environmental contamination has not been investigated fully. Sewage treatment plants (STPs) are one of the most important interfaces between the human population and the aquatic environment. Several previous studies (3–5) have proposed STPs and wastewater to be the hot spots for horizontal gene transfer, facilitating the spread of antimicrobial resistance (AMR) genes, including carbapenemase genes, between different bacterial species. Additionally, sewerage system diffusion has improved in recent years, leading to an increased proportion of sewage effluent in the environmental water. These facts suggest that STPs and wastewater can act as anthropogenic sources, reservoirs, and environmental suppliers of AMR genes.

A large variety of carbapenemases have been reported, including those belonging to Ambler class A (e.g., KPC and IMI), class B (e.g., IMP and NDM), and class D (e.g., OXA-48 and OXA-162) (6). In clinical fields, highly carbapenem-resistant strains harboring KPC- or NDM-producing *Enterobacteriaceae* have been spreading rapidly between countries (7, 8). In Japan, the most prevalent carbapenemase in *Enterobacteriaceae* is an IMP-type enzyme (9, 10). On the other hand, NDM-, VIM-, KPC-, or OXA-48-producing *Enterobacteriaceae* have been rarely isolated from sporadic cases (e.g., patients with carbapenem-resistant infections who have travelled abroad) (8, 11). Ambler class B carbapenemases are metallo-β-lactamases (MBLs) and classified into various types according to their amino acid sequences. In general, MBLs harbor hydrolytic activity against broad-spectrum β-lactams except monobactams and demonstrate reduced carbapenem susceptibility (12). Kyorin Health Science MBL-1 (KHM-1) was identified in 1997 in a multidrug-resistant Citrobacter freundii isolate from a patient with a catheter-associated urinary tract infection, in Japan. The *bla*_KHM-1_ gene was carried in a plasmid of approximately 200 kbp, designated pCF243 (13). However, reappearance of this enzyme has not been identified in clinical settings or the natural environment since its first report (14). Thus, the extent of spread and whether this MBL can contribute to CPE infections are unknown.

We reported the isolation of a novel multidrug-resistant IncA/C2 plasmid, pTMSNI47-1, containing carbapenemase gene *bla*_KHM-1_, in Klebsiella quasipneumoniae SNI47 isolated from a municipal STP in Japan. It was suggested that CPE, harboring a highly transferable and broad-spectrum resistance plasmid, had been disseminated and deposited into the sewage.

## RESULTS

### Detection of carbapenem-resistant Klebsiella quasipneumoniae isolate SNI47 in urban sewage.

**FIG 1:**
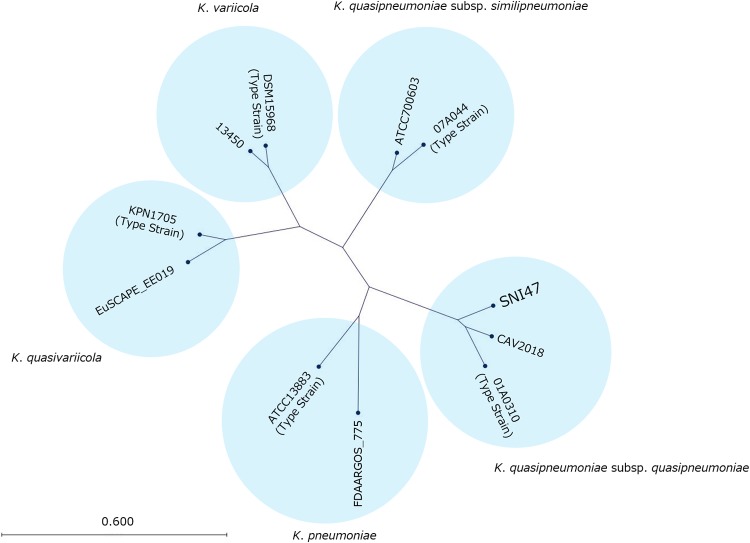
Nucleotide divergence of Klebsiella pneumoniae, K. variicola, K. quasivariicola, K. quasipneumoniae subsp. *quasipneumoniae*, and K. quasipneumoniae subsp. *similipneumoniae* strains. The neighbor-joining tree was generated on the basis of the k-mer distribution among SNI47, Klebsiella pneumoniae strains ATCC 13883 and FDAARGOS_775, K. variicola strains DSM15968 and 13450, K. quasivariicola strains KPN1705 and EuSCAPE_EE019, K. quasipneumoniae subsp. *quasipneumoniae* strains 01A030 and CAV2018, and K. quasipneumoniae subsp. *similipneumoniae* strains 07A044 and ATCC 700603.

**FIG 2:**
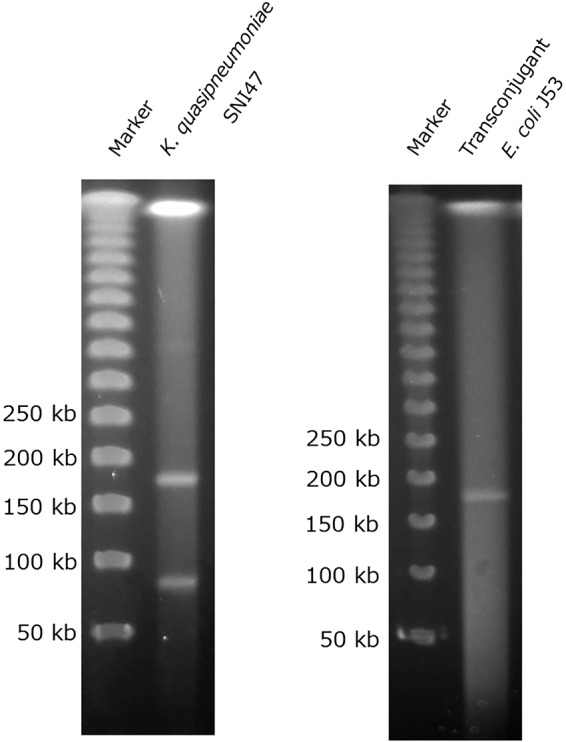
Pulsed-field gel electrophoresis (PFGE) of S1 nuclease-digested total DNA plugs. (Left) S1-PFGE pattern observed in Klebsiella quasipneumoniae subsp. *quasipneumoniae* SNI47; (right) S1-PFGE pattern observed in transconjugant Escherichia coli J53. A lambda ladder (Promega, Fitchburg, WI) was used as the size marker.

We initially used a short-read next-generation sequencer (MiSeq) to confirm the phylogenetic position of the carbapenem-resistant isolate SNI47 relative to typical strains of Klebsiella pneumoniae, Klebsiella variicola, Klebsiella quasivariicola, Klebsiella quasipneumoniae subsp. *quasipneumoniae*, and Klebsiella quasipneumoniae subsp. *similipneumoniae*. Average nucleotide identity (ANI) analysis showed that the nucleotide sequence of SNI47 was similar (99.18%) to that of 01A030 strain, a type strain of Klebsiella quasipneumoniae subsp. *quasipneumoniae*. In addition, the resultant tree, based on k-mer diversity, indicated that the genomic structure of SNI47 was quite similar to that of K. quasipneumoniae subsp. *quasipneumoniae* strains (Fig. 1). S1 pulsed-field gel electrophoresis (S1-PFGE) analysis for SNI47 revealed two bands, approximately 180 kbp and 80 kbp (Fig. 2, left). MiSeq analysis for gel-extracted chromosome or plasmid DNA fragments revealed that the AMR determinants located on the chromosome were *bla*_OKP-A-4_ (conferring reduced susceptibility to β-lactams), *oqxA* and *oqxB* (quinolones), and *fosA* (fosfomycin). An approximately 180-kbp DNA fragment carried IncA/C2 and IncFIB(K) replicons and 10 types of AMR determinants, namely, *aadA1* (aminoglycosides); *bla*_CTX-M-2,_ *bla*_DHA-1,_ *bla*_KHM-1_, and *bla*_OXA-10_ (β-lactams); *qnrB4* (quinolones); *cmlA5* (chloramphenicol); *arr2* (rifampin); *sul1* (sulfonamide); and *dfrA14* (trimethoprim). IncFII(K) and IncR replicons were detected in the approximately 80-kbp DNA fragment; however, AMR genes were absent.

**FIG 3:**
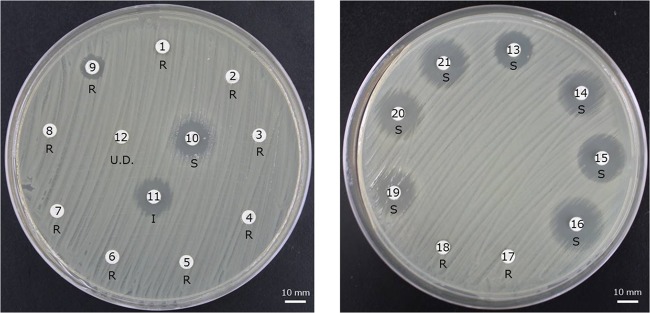
Sizes of zones of inhibition of Klebsiella quasipneumoniae subsp. *quasipneumoniae* SNI47 by 21 antimicrobials in the Kirby-Bauer drug susceptibility test. The following antimicrobials were tested: 1, imipenem; 2, meropenem; 3, penicillin; 4, ampicillin; 5, cefotaxime; 6, ceftazidime; 7, cefoxitin; 8, cephalothin; 9, aztreonam; 10, fosfomycin; 11, chloramphenicol; 12, rifampin; 13, nalidixic acid; 14, norfloxacin; 15, levofloxacin; 16, ciprofloxacin; 17, sulfamethoxazole-trimethoprim; 18, streptomycin; 19, kanamycin; 20, gentamicin; and 21, amikacin. Susceptibility criteria conformed to the Clinical and Laboratory Standards Institute guidelines. S, susceptible; I, intermediate; R, resistant; U.D., susceptibility undetermined because interpretative criteria were not defined for *Enterobacteriaceae*.

Antibiotic susceptibility testing using BD Sensi-Discs revealed that K. quasipneumoniae subsp. *quasipneumoniae* SNI47 was resistant to all tested β-lactams, rifampin, streptomycin, and sulfamethoxazole-trimethoprim (SXT) and sensitive to fosfomycin, kanamycin, gentamicin, amikacin, and all tested quinolones. Intermediate susceptibility to chloramphenicol was shown (Fig. 3). MICs of the β-lactams were 24 μg/ml for aztreonam, >32 μg/ml for imipenem, meropenem, ertapenem, doripenem, benzylpenicillin, and cefotaxime, and >256 μg/ml for amoxicillin, piperacillin, ceftazidime, and cefepime (Table 1). MICs of the aminoglycosides and fluoroquinolones were 32, 1, 2, 0.5, and 1 μg/ml for streptomycin, gentamicin, amikacin, ciprofloxacin, and levofloxacin, respectively (Table 2).

**TABLE 1:** Etest results of β-lactams on Klebsiella quasipneumoniae subsp. *quasipneumoniae* SNI47, Escherichia coli J53, and Escherichia coli DH5α transconjugant and transformants

Antimicrobial	MIC (μg/ml)										
	SNI47	E. coli									
		J53	J53/pTMSNI47-1	DH5α	DH5α/pHSG398	DH5α/pHSG-*bla*_CTX-M-2_	DH5α/pHSG-*bla*_DHA-1_	DH5α/pHSG-*bla*_KHM-1_	DH5α/pHSG-*bla*_OXA-10_	DH5α/pHSG-*bla*_OKP-A-4_	DH5α/pTMSNI47-1
Imipenem	>32	0.125	4	0.125	0.125	0.25	0.125	0.5	0.25	0.125	4
Meropenem	>32	≤0.06	2	≤0.06	≤0.06	≤0.06	≤0.06	0.5	≤0.06	≤0.06	2
Ertapenem	>32	≤0.06	2	≤0.06	≤0.06	≤0.06	≤0.06	0.25	≤0.06	≤0.06	2
Doripenem	>32	0.125	4	≤0.06	≤0.06	0.125	0.125	0.25	0.125	≤0.06	4
Benzylpenicillin	>32	>32	>32	>32	>32	>32	>32	>32	>32	>32	>32
Amoxicillin	>256	8	>256	4	4	>256	>256	64	>256	>256	>256
Piperacillin	>256	1	>256	0.5	0.5	>256	32	1	>256	>256	>256
Ceftazidime	>256	0.125	>256	0.125	0.125	0.5	4	>256	0.125	0.125	>256
Ceftazidime-clavulanic acid	>4	0.125	>4	≤0.125	≤0.125	≤0.125	>4	>4	≤0.125	≤0.125	>4
Cefotaxime	>32	≤0.06	>32	≤0.06	≤0.06	>32	2	16	0.125	≤ 0.06	>32
Cefotaxime-clavulanic acid	>1	≤0.06	>1	≤0.06	≤0.06	≤0.06	>1	>1	≤0.06	≤0.06	>1
Cefepime	>256	≤0.06	64	≤0.06	≤0.06	8	≤0.06	4	0.125	≤0.06	64
Cefepime-clavulanic acid	>4	≤0.125	>4	≤0.125	≤0.125	0.125	≤0.125	>4	≤0.125	≤0.125	>4
Aztreonam	24	≤0.06	4	≤0.06	≤0.06	8	1	≤0.06	0.25	≤0.06	2

**TABLE 2:** Etest results of aminoglycosides and fluoroquinolones on Klebsiella quasipneumoniae subsp. *quasipneumoniae* SNI47, Escherichia coli J53, and transconjugant J53/pTMSNI47-1

Antimicrobial	MIC (μg/ml)		
	SNI47	E. coli J53	E. coli J53/pTMSNI47-1
Streptomycin	32	1	8
Gentamicin	1	≤0.06	≤0.06
Amikacin	2	0.5	0.5
Ciprofloxacin	0.5	≤0.06	0.25
Levofloxacin	1	≤0.06	0.5

### Transmission ability of pTMSNI47-1 and antimicrobial susceptibility of transconjugant.

A conjugation experiment was performed with SNI47 and Escherichia coli J53. Transconjugants that were resistant to both sodium azide and ampicillin were obtained. According to S1-PFGE, the transconjugants contained an approximately 180-kbp plasmid (Fig. 2, right). The transconjugants exhibited a susceptibility profile similar to that of K. quasipneumoniae subsp. *quasipneumoniae* SNI47, although the MICs for carbapenems (imipenem, 4 μg/ml; meropenem, 2 μg/ml; ertapenem, 2 μg/ml; and doripenem, 4 μg/ml), cefepime (64 μg/ml), and aztreonam (4 μg/ml) were lower in the transconjugants than in SNI47 (Table 1).

### Antimicrobial susceptibilities of five β-lactamases.

To detect which β-lactamases harbored hydrolytic activity against each β-lactam, we transformed five recombinant plasmids to DH5α cells and measured the MICs of 10 β-lactams. As Table 1 shows, both CTX-M-2-producing and DHA-1-producing strains showed increased MICs of penicillin, cephem, or monobactam derivatives. In particular, the MICs of cefotaxime (>32 μg/ml), cefepime (8 μg/ml), and aztreonam (8 μg/ml) for the former were the largest among the five tested β-lactamases. The production of CTX-M-2 had a small effect on the hydrolytic activity against ceftazidime (0.5 μg/ml). On the other hand, the DHA-1-producing strain showed a higher MIC increase for ceftazidime (4 μg/ml) and lower MIC increases for piperacillin (32 μg/ml), cefotaxime (2 μg/ml), and aztreonam (1 μg/ml) than those of the CTX-M-2-producing strain. Regarding the three cephem derivatives, clavulanic acid could inhibit CTX-M-2 activities but could not inhibit DHA-1. The KHM-1-producing strain showed generally increased MICs of carbapenems and cephems. The MICs of carbapenems were 0.5 μg/ml for imipenem, 0.5 μg/ml for meropenem, 0.25 μg/ml for ertapenem, and 0.25 μg/ml for doripenem, corresponding to 4-, >8-, >4-, and >4-fold increases compared with those for the control DH5α strain, respectively. As expected, the production of KHM-1 had no effect on the MIC of aztreonam. The production of OXA-10 increased the MICs of amoxicillin (>256 μg/ml) and piperacillin (>256 μg/ml). The production of chromosomal β-lactamase, OKP-A-4, only increased the MICs of penicillin derivatives. DH5α transformed with pTMSNI47-1 exhibited a susceptibility profile almost identical to that of transconjugant J53/pTMSNI47-1.

### Whole-genome sequencing of SNI47.

The hybrid assembly of MiSeq and MinION reads gave one complete chromosome sequence (5,468,267 bp) and four complete plasmid sequences over 50 kbp, namely, 185,311 bp (pTMSNI47-1), 181,469 bp (pTMSNI47-2), 89,834 bp (pTMSNI47-3), and 85,849 bp (pTMSNI47-4). The sequence type (ST) for the chromosome was ST668. The incompatibility (Inc) type and sequence type were as follows: IncA/C2 and ST26 (pTMSNI47-1), IncFIB(K) and not available (pTMSNI47-2), IncFII(K) and allele identifier (ID) 21 (pTMSNI47-3), and IncR and not available (pTMSNI47-4). AMR genes were not found on pTMSNI47-2, pTMSNI47-3, or pTMSNI47-4 (Table 3). These results were consistent with those of S1-PFGE and MiSeq analysis using the gel-extracted DNA samples.

**TABLE 3:** Whole-genome information for Klebsiella quasipneumoniae subsp. *quasipneumoniae* SNI47 isolated from influent water from a sewage treatment plant in Japan

Replicon	GC (%)	Length (bp)	Incompatibility group	Sequence type and/or allele ID	Drug resistance gene(s)	Resistance phenotype
Chromosome	57.80	5,468,267		ST668	*bla*_OKP-A-4_	β-Lactams
					*oqxA*, *oqxB*	Quinolones
					*fosA*	Fosfomycin
pTMSNI47-1	51.24	185,311	IncA/C2	ST26, cgST26.1	*aadA1*	Aminoglycosides
					*bla*_CTX-M-2_, *bla*_DHA-1_, *bla*_KHM-1_, *bla*_OXA-10_	β-Lactams
					*qnrB4*	Quinolones
					*cmlA5*	Chloramphenicol
					*arr2*	Rifampin
					*sul1*	Sulfonamides
					*dfrA14*	Trimethoprim
pTMSNI47-2	50.37	181,469	IncFIB(K)	Not available	Not found	
pTMSNI47-3	53.02	89,843	IncFII(K)	Allele ID 21	Not found	
pTMSNI47-4	50.13	85,849	IncR	Not available	Not found	

**FIG 4:**
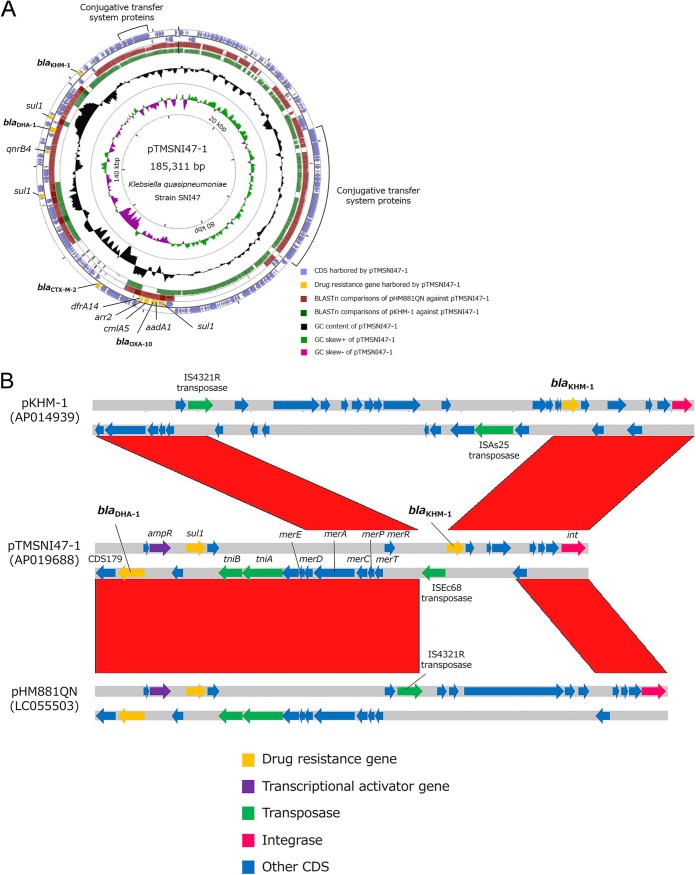
Genome structure of pTMSNI47-1. (A) Genome representation was performed using CGview Server. The outermost two rings comprising coloured arrowheads show features extracted from the pTMSNI47-1 genome. Yellow arrowheads show drug resistance genes harbored by pTMSNI47-1. The next three rings show the positions of BLAST hits detected through BLASTn comparisons of the pTMSNI47-1 genome against the two plasmid genomes (pHM881QN and pKHM-1, represented by red and green circles, respectively). Darker arcs indicate a high percent identity of the hit. The black circle displays the GC content, and inner circles display GC skew. (B) Comparison of the structures among pTMSNI47-1, pHM881QN, and pKHM-1. Comparisons were performed using the Artemis Comparison Tool with a minimum score cutoff 200 and a minimum percent identity cutoff of 80%. Forward matches are colored in red.

pTMSNI47-1 displayed overall constant levels of homology with other IncA/C2 plasmids found in K. pneumoniae (pHM881QN, GenBank accession number LC055503, 71% query cover) and in Citrobacter freundii (pCFJY-17, GenBank accession number MH763829, 71% query cover). The 223 coding DNA sequences (CDSs) were annotated in pTMSNI47-1, and the genes that contributed to conjugal transfer, such as *traA*, *traB*, and *traC*, were detected in two regions of the pTMSNI47-1 sequence (Fig. 4A). Ten AMR genes, which were identified using ResFinder, were detected in these CDSs, namely, *aadA1* (CDS125), *bla*_CTX-M-2_ (CDS136), *bla*_DHA-1_ (CDS180), *bla*_KHM-1_ (CDS197), *bla*_OXA-10_ (CDS126), *qnrB4* (CDS174), *cmlA5* (CDS127), *arr2* (CDS128), and *dfrA14* (CDS129). *sul1* was triplicated in pTMSNI47-1 (CDS123, CDS164, and CDS184). Analysis of the genetic context revealed insertion sequence (IS) transposase-encoding or recombinase/integrase-encoding genes in the vicinity of *bla*_CTX-M-2_, *bla*_DHA-1_, and *bla*_KHM-1_ (Fig. 4B), namely, genes for an IS*1380*-like element ISEc9 family transposase (CDS135), IS*5*-like element ISEc68 family transposase (CDS196), Tn*3* family transposase (CDS133, CDS134, CDS186, and CDS187), and tyrosine-type recombinase/integrase (CDS205). pTMSNI47-1 carried two distinct class 1 integrons. One was located between CDS162 (*intl1*) and CDS164 (*sul1*) with an empty gene cassette region. The other was located complementarily in the region between CDS123 (*sul1*) and CDS130 (*intl1*) and comprised the *aadA1*-*bla*_OXA-10-_*cmlA5*-*arr2*-*dfrA14* gene cassette array. This sequence was identical to that of In633 harbored by a Providencia stuartii strain PsB/3 plasmid (GenBank accession number JN193567).

## DISCUSSION

STPs are considered major anthropogenic sources, reservoirs, and environmental suppliers of AMR genes, including carbapenemase genes (3, 4). In fact, recent studies of Japanese STPs identified GES-5, GES-24, IMP-19, and KPC-2-type CPE isolates from wastewater (11, 15). Our study identified a CPE isolate, SNI47, from the influent water of an STP in Japan. The phylogenetic tree constructed using MiSeq data illustrated that SNI47 belongs to K. quasipneumoniae subsp. *quasipneumoniae* (Fig. 1). In clinical settings, a higher prevalence of K. pneumoniae than K. quasipneumoniae has been reported (16, 17). Gomi et al. (15) reported that the prevalence among the carbapenemase-producing *Klebsiella* isolates from STPs or hospital wastewater was different from those observed in clinical isolates, with K. quasipneumoniae isolated more frequently than K. pneumoniae from wastewater. Our results support their suggestion that the difference may primarily be because K. quasipneumoniae is associated more frequently with carriage, whereas K. pneumoniae is associated with human infection.

K. quasipneumoniae subsp. *quasipneumoniae* SNI47 harbored four plasmids: IncA/C2 plasmid pTMSNI47-1, IncFIB(K) plasmid pTMSNI47-2, IncFII(K) plasmid pTMSNI47-3, and IncR plasmid pTMSNI47-4. Of these, only pTMSNI47-1 harbored drug resistance genes (Table 3). IncA/C plasmids have demonstrated a wide *Enterobacteriaceae* host range and are one of the main plasmid families that mediate AMR dissemination (18). In addition, our whole-genome sequencing results indicated that the genes contributing to conjugal transfer, such as *traA*, were annotated in pTMSNI47-1 (Fig. 4A). It was demonstrated experimentally that pTMSNI47-1 could transfer among *Enterobacteriaceae* through conjugation. The MICs of all tested β-lactams, including carbapenems, for both transconjugant J53/pTMSNI47-1 and transformants DH5α/pTMSNI47-1 were higher than those for the recipient strain, indicating that this plasmid conferred resistant properties for carbapenems. The broad host range and the high self-transferability of plasmid pTMSNI47-1 can lead to drug resistance acquisition in one horizontal gene transfer event.

Gene module transpositions are facilitated by transposons and ISs. These elements are frequently detected in plasmids, including pTMSNI47-1, that involve AMR genes, non-AMR genes, and transposable genetic elements. For example, analysis of the genetic context showed ISEc68 and recombinase/integrase-encoding genes in the vicinity of *bla*_KHM-1_. The ISEc68 transposase gene was flanked by 17-bp inverted repeats IRL (5′-GGAAGGTGCGAATAAGT-3′) and IRR (5′-ACTTAATCGCAGCTTCC-3′); however, the repeat regions did not encompass the KHM-1-encoding gene. On the other hand, the region from *bla*_KHM-1_ to *int*, encoding tyrosine-type integrase, was conserved (Fig. 4B). It is possible that both plasmids acquired this region from a common ancestor (i.e., *bla*_KHM-1_-harboring mobile genetic element) via homologous recombination in the evolution process. Further studies are required to conclude which elements mediate a transposition mechanism involving *bla*_KHM-1_. In addition, *bla*_OXA10_ was located in a class 1 integron, which includes a site-specific recombination system capable of integrating and expressing open reading frames contained in structures called mobile gene cassettes. This integron gene cassette array, *aadA1*-*bla*_OXA-10-_*cmlA5*-*arr2*-*dfrA14*, has been identified in other plasmids harbored by several *Enterobacteriaceae*, such as Providencia stuartii (19), demonstrating that gene module transpositions are a vital factor in the successful spread of *bla* genes among various plasmids.

An increase in MICs of β-lactams was detected with transformants of E. coli DH5α with the recombinant plasmids pHSG-bla_CTX-M-2_, pHSG-bla_DHA-1_, pHSG-bla_KHM-1_, pHSG-bla_OXA-10_, and pHSG-bla_OKP-A-4_. Of these, *bla*_CTX-M-2_ enhanced amoxicillin, piperacillin, cefotaxime, cefepime, and aztreonam resistance, while the *bla*_DHA-1_ strain showed a higher MIC increase for ceftazidime than the *bla*_CTX-M-2_ stain. Clavulanic acid, a mechanism-based β-lactamase inhibitor, could inhibit CTX-M-2 activities but could not inhibit DHA-1 (Table 1). The production of OXA-10 or OKP-A-4 strongly increased the MICs of amoxicillin and piperacillin (Table 1). CTX-M-type enzymes are classified as Ambler class A extended-spectrum β-lactamases (ESBLs) and exhibit a striking substrate preference for cefotaxime and ceftriaxone over ceftazidime because of the unique geometry of the β-lactam-binding site (20). DHA-1 is an AmpC-type β-lactamase belonging to Ambler class C and generally not inhibited by clavulanic acid. AmpC-type β-lactamases harbor hydrolytic activity against cephalosporin and cefamycin and variably to aztreonam, but they remain sensitive to cefepime and carbapenems (21, 22). OXA-10-type enzyme, classified as Ambler class D, was known to have narrow-spectrum β-lactamase activity, although variants in this enzyme family (such as OXA-11 and OXA-16) exhibit expanded-spectrum activity (23). The class A β-lactamase OKP enzymes, which are similar to SHV and LEN enzymes, exist in almost all K. quasipneumoniae chromosomes and are penicillinases (24, 25). Our antimicrobial susceptibility results are completely consistent with those of these previous studies.

Only *bla*_KHM-1_ enhanced carbapenem resistance (Table 1). KHM-1 is an acquired Ambler class B MBL and harbors the hydrolytic efficiencies of carbapenems. So far, this enzyme has been recognized as a rare form of MBL because there is only one report of its isolation (14). However, our study has isolated KHM-1 in a transferable plasmid (pTMSNI47-1) harbored by an efficient carrier (K. quasipneumoniae) from a hot spot for horizontal gene transfer (an STP), and this enzyme might be widely disseminated in the near future in Japan. Carbapenem resistance has been documented by several mechanisms besides carbapenemase production. One of them is the loss of outer membrane porins combined with ESBLs or AmpC β-lactamases (26, 27). We did not examine the expression of porins, CTX-M-2, or DHA-1 in this study; therefore, these may also have influenced carbapenem resistance. However, regardless of the mechanism of resistance, horizontal transfer of pTMSNI47-1, harboring four different Ambler class β-lactamases, leads to a very-wide-spectrum β-lactam resistance acquisition with mutual compensation.

Except for β-lactams, SNI47 was fully resistant to SXT (Fig. 3). Previous reports have stated that *sul* contributes to SXT resistance, and the *sul* and *dfrA* genes could synergistically lead to high-level SXT resistance (28, 29). Both genes exist in pTMSNI47-1. Additionally, it was interesting to find that the *sul1* gene was triplicated in this plasmid (Fig. 4A), suggesting that these genes, especially triplicated *sul1* genes, might play an important role in high-level SXT resistance. On the other hand, SNI47 was sensitive to kanamycin, gentamicin, and amikacin (Fig. 3). Aminoglycoside resistance may occur due to several mechanisms. Of them, *aadA1*, which is located in the class 1 integron of pTMSNI47-1, encodes an aminoglycoside adenylyltransferase that influences enzymatic modification and inactivation (30). This enzyme imparts streptomycin and spectinomycin resistance by modifying the 3-hydroxyl position of streptomycin and the 9-hydroxyl position of spectinomycin, but it has marginal effects on the resistance to other aminoglycosides (30). Our results are consistent with those of these previous studies (Fig. 3 and Table 2).

The *oqxA* and *oqxB* genes, encoding the OqxAB efflux pump, and plasmid-mediated quinolone resistance (PMQR) gene *qnrB4* were found in the chromosomes of SNI47 and pTMSNI47-1, respectively. However, contrary to our expectations, SNI47 was sensitive to all tested quinolones (Fig. 3) and did not reach the Clinical and Laboratory Standards Institute (CLSI) breakpoint (Table 2). At least three distinct mechanisms of quinolone resistance in bacteria have been identified: (i) mutations in target enzymes, (ii) alteration in membrane permeability, and (iii) protection of target enzymes from quinolone inhibition (31). Of them, resistance is mediated mainly by the accumulation of point mutations in the quinolone resistance-determining region (QRDR) of DNA gyrase (*gyrA*) and DNA topoisomerase IV (*parC*) (3, 31). It was reported that differences in the expression level of OqxAB influenced reduced susceptibility to quinolones; however, most fell short of susceptibility breakpoints (32). PMQR genes contribute to the low level of resistance to fluoroquinolones, and these genes exert their influence by widening the mutant selection window and elevating mutant prevention (33). Our present Etest results for fluoroquinolones support the abovementioned contention. The MICs for SNI47, which harbored the *oqxA*, *oqxB*, and *qnrB4* genes and did not harbor mutations in QRDR, were 0.5 μg/ml for ciprofloxacin and 1 μg/ml for levofloxacin. On the other hand, those for transconjugant J53/pTMSNI47-1 (harboring only *qnrB4*) were 0.25 μg/ml for ciprofloxacin and 0.5 μg/ml for levofloxacin, corresponding to >4- and >8-fold increases compared with those for the recipient strain, respectively (Table 2). To some extent, these genes contribute to fluoroquinolone resistance, although both SNI47 and transconjugants J53/pTMSNI47-1 did not reach the CLSI breakpoint, even for intermediate resistance.

The present study uncovered evidence that the horizontal transfer of pTMSNI47-1 was a causative agent of a very-wide-spectrum β-lactam resistance. Whole-genome sequencing illustrated that isolate SNI47 was K. quasipneumoniae subsp. *quasipneumoniae* and harbored four plasmids. Of these, the IncA/C2 plasmid, pTMSNI47-1, could transfer to E. coli J53 through conjugation and carried resistance genes, including those for four β-lactamases (*bla*_CTX-M-2_, *bla*_DHA-1_, *bla*_KHM-1_, and *bla*_OXA-10_). The MICs of β-lactams for both transconjugant J53/pTMSNI47-1 and transformant DH5α/pTMSNI47-1 were higher than those for the recipient strain. Furthermore, *bla*_KHM-1_ was mainly responsible for reducing carbapenem susceptibility. Our findings indicate that this plasmid conferred properties for wide-spectrum β-lactam resistance, including carbapenems, with mutual compensation. The highly transferable plasmid pTMSNI47-1 was detected in efficient carriers isolated from hot spots for horizontal gene transfer. Therefore, it might be widely disseminated and become a concerning clinical issue in the near future.

## MATERIALS AND METHODS

### Bacterial strains used in this study.

A 10-ml aliquot of influent water from an STP in Japan was cultured overnight in 2× brilliant green lactose bile (BGLB) broth (Eiken Chemical, Tokyo, Japan) at 37°C. A 1-ml aliquot of the gas-producing BGLB broth culture was spread on a Pro-media Tricolor agar (ELMEX, Tokyo, Japan) plate to perform antimicrobial susceptibility testing with imipenem and meropenem using BD Sensi-Discs (Becton, Dickinson and Company, Franklin Lakes, NJ). Red colonies (β-galactosidase produced by coliforms hydrolyzes the Magenta-GAL complex) formed inside the inhibition rings were picked, and we determined the MICs of imipenem and meropenem using the dry-strip technique (Etest; bioMérieux, La Balme-les-Grottes, France) according to CLSI criteria. MICs of >32 mg/liter were identified for each drug. We designated this coliform isolate strain SNI47.

### S1 pulsed-field gel electrophoresis and gel extraction.

S1-PFGE, for separating plasmids from chromosomes, was performed according to the method of Barton et al. (34), with modifications. Briefly, DNA plugs digested with S1 nuclease (Takara Bio, Shiga, Japan) were electrophoresed on a CHEF Mapper XA PFGE system (Bio-Rad, Hercules, CA) with autoalgorithms for 5 to 250 kbp. A lambda ladder (Promega, Fitchburg, WI) was used as the size marker. Extraction of the chromosome or plasmid DNA fragments from the electrophoresed gel for MiSeq analysis was performed using a Zymoclean large-fragment DNA recovery kit (Funakoshi Co., Ltd., Tokyo, Japan).

### Preparation of genomic DNA.

The genomic DNA of SNI47 was extracted for MiSeq analysis using a QIAamp DNA minikit (Qiagen GmbH, Hilden, Germany). The concentration of the extracted genomic DNA was determined using a QuantiFluor ONE double-stranded DNA (dsDNA) system (Promega), and its purity was assessed using a NanoDrop 2000c spectrophotometer (Thermo Fisher Scientific, Waltham, MA).

### DNA library preparation and MiSeq analysis.

An index-tagged library was prepared using the Nextera XT DNA library preparation kit (Illumina, San Diego, CA), and 300-bp paired-end reads were sequenced on an Illumina MiSeq instrument according to the manufacturer’s instructions. To ensure that only high-quality data were used for assembly, reads were trimmed and filtered using the CLC Genomics Workbench 11.0 (Qiagen) set to a minimum length of 100 bp and a quality score threshold of 30. These trimmed reads were assembled *de novo* using the CLC Genomics Workbench with default settings. Species identification was performed by ANI analysis (http://enve-omics.ce.gatech.edu/ani/). Phylogenetic analysis of k-mer diversity was performed according to a previously reported method (35). The k-mer length was set at 16 bases for this analysis. The MLST web server (https://cge.cbs.dtu.dk/services/MLST/) was used to determine sequence types for chromosome. The PubMLST web server (https://pubmlst.org/) was used to determine sequence types or allele ID for plasmid. The ResFinder web server (https://cge.cbs.dtu.dk/services/ResFinder/) was used to identify AMR genes. The PlasmidFinder web server (https://cge.cbs.dtu.dk/services/PlasmidFinder/) was used to identify the Inc type. The threshold for minimum coverage was set at 60% of the length of the gene sequence in the database, with a minimum sequence identity of 80%.

### Antimicrobial susceptibility testing.

A Kirby-Bauer disc diffusion test was performed for SNI47 using BD Sensi-Discs and Mueller-Hinton agar plates (Becton, Dickinson and Company) according to CLSI recommendations. The following antimicrobials were tested: imipenem (10 μg), meropenem (10 μg), penicillin (30 μg), ampicillin (10 μg), cefotaxime (30 μg), ceftazidime (30 μg), cefoxitin (30 μg), cephalothin (30 μg), aztreonam (30 μg), fosfomycin (50 μg), chloramphenicol (30 μg), rifampin (5 μg), nalidixic acid (30 μg), norfloxacin (10 μg), levofloxacin (5 μg), ciprofloxacin (5 μg), SXT (23.75/1.25 μg), kanamycin (30 μg), gentamicin (10 μg), and amikacin (30 μg). Then the MICs of four carbapenem derivatives (imipenem, meropenem, ertapenem, and doripenem), two penicillin derivatives (amoxicillin and piperacillin), three cephem derivatives (ceftazidime, cefotaxime, and cefepime), one monobactam derivative (aztreonam), ceftazidime-clavulanic acid, cefotaxime-clavulanic acid, and cefepime-clavulanic acid were determined using the dry-strip technique (Etest) according to the guidelines of the CLSI.

### Conjugation experiment.

The SNI47 strain containing a β-lactamase-encoding plasmid was used as a donor, and the spontaneous-sodium azide-resistant E. coli J53 strain was used as a recipient in our conjugation experiment. The recipient was susceptible to all antibiotics tested and did not harbor a β-lactamase-encoding plasmid. SNI47 and E. coli J53 were mixed in a 1:1 ratio, and then the mixture was cultured at 37°C for 5 h. Transconjugants were selected on Pro-media tricolor agar supplemented with sodium azide (100 μg/ml) and ampicillin (50 μg/ml). The blue colonies (β-glucuronidase produced by E. coli hydrolyzes the 5-bromo-4-chloro-3-indolyl-β-d-glucuronic acid [X-Gluc] complex, indicating successful plasmid transformation) were picked and subjected to S1-PFGE. Then plasmid DNA was extracted from the transconjugant colonies using a HiSpeed plasmid maxikit (Qiagen). E. coli DH5α cells were transformed with the extracted plasmid. The Etest dry-strip technique was used to determine the hydrolytic activities of the J53 transconjugants and DH5α transformants.

### Hydrolytic activities for β-lactams by exogenous genes.

Tables 4 and 5 present plasmids and the primers used for plasmid construction. The *bla*_CTX-M-2_, *bla*_DHA-1_, *bla*_KHM-1_, *bla*_OXA-10_, and *bla*_OKP-A-4_ fragments were amplified by PCR using KOD-Plus-Ver. 2 polymerase (Toyobo, Osaka, Japan), and the PCR products were directly cloned into a pHSG398 vector (Takara Bio) using an In-Fusion HD cloning kit (Takara Bio). The resulting vectors were designated pHSG-bla_CTX-M-2_, pHSG-bla_DHA-1_, pHSG-bla_KHM-1_, pHSG-bla_OXA-10_, and pHSG-bla_OKP-A-4_ (Table 5). Nucleotide sequences were verified using an ABI 3130 capillary sequencer (Thermo Fisher Scientific) with a BigDye Terminator v3.1 cycle sequencing kit (Thermo Fisher Scientific). E. coli DH5α cells were transformed with each cloning plasmid to evaluate the expression and the substrate specificity of each exogenous β-lactamase. The Etest dry-strip technique was used to determine their hydrolytic activities.

**TABLE 4:** Nucleotide sequences used for cloning in this study

Gene	Name	Oligonucleotide sequence (5′→3′)
*bla*_CTX-M-2_	CTXM2_IF_F	CATGATTACGAATTCGATGACTCAGAGCAT
	CTXM2_IF_R	GGCCAGTGCCAAGCTTCAGAAACCGTGGGT
*bla*_DHA-1_	DHA1_IF_F	CATGATTACGAATTCGAAAAAATCGTTATC
	DHA1_IF_R	GGCCAGTGCCAAGCTTTATTCCAGTGCACT
*bla*_KHM-1_	KHM1_IF_F	CATGATTACGAATTCGAAAATAGCTCTTGT
	KHM1_IF_R	GGCCAGTGCCAAGCTTCACTTTTTAGCTGC
*bla*_OXA-10_	OXA10_IF_F	CATGATTACGAATTCGAAAACATTTGCCGC
	OXA10_IF_R	GGCCAGTGCCAAGCTTTAGCCACCAATGAT
*bla*_OKP-A-4_	OKPA4_IF_F	CATGATTACGAATTCGCGTTATGTTCGCCT
	OKPA4_IF_R	GGCCAGTGCCAAGCTCTAGCGCTGCCAGTG

**TABLE 5:** Plasmids used in this study

Plasmid	Relevant characteristics	Source
pHSG398	Cm^r^, cloning vector	Takara
pHSG-bla_CTX-M-2_	Cm^r^; pHSG398 with the cloned PCR product containing *bla*_CTX-M-2_ full-length sequence	This study
pHSG-bla_DHA-1_	Cm^r^; pHSG398 with the cloned PCR product containing *bla*_DHA-1_ full-length sequence	This study
pHSG-bla_KHM-1_	Cm^r^; pHSG398 with the cloned PCR product containing *bla*_KHM-1_ full-length sequence	This study
pHSG-bla_OXA-10_	Cm^r^; pHSG398 with the cloned PCR product containing *bla*_OXA-10_ full-length sequence	This study
pHSG-bla_OKP-A-4_	Cm^r^; pHSG398 with the cloned PCR product containing *bla*_OKP-A-4_ full-length sequence	This study

### Whole-genome sequencing using Illumina MiSeq and Oxford Nanopore MinION.

The complete genome sequence of SNI47 was obtained by combining sequencing data from both Illumina Miseq and MinION (Oxford Nanopore Technologies, Oxford, UK) sequencers. Illumina sequencing was performed as described above, and Nanopore sequencing was performed according to the manufacturer’s instructions. Briefly, a DNA library was prepared using a rapid sequencing kit (Oxford Nanopore Technologies), and the prepared library was subsequently loaded into a MinION flow cell (R9.4; Oxford Nanopore Technologies). The MinION sequencing run was performed over 48 h. Hybrid assembly of both the MiSeq and MinION reads was performed using Unicycler v0.4.2 (36) with default settings and annotated using Prokka v1.12 (37) and DFAST (https://dfast.nig.ac.jp/). Further annotation was performed manually using information from National Center for Biotechnology Information BLASTn for any unknown CDSs. The annotation of ISs was performed using ISFinder (https://isfinder.biotoul.fr/). When complete, the genome of pTMSNI47-1 was viewed using CGview Server (http://stothard.afns.ualberta.ca/cgview_server/), and a comparison of the regions of interest was facilitated using the Artemis Comparison Tool (38).

### Data availability.

The DDBJ accession numbers for sequences obtained in this study are as follows: AP019687 (SNI47 chromosome), AP019688 (pTMSNI47-1), AP019689 (pTMSNI47-2), AP019690 (pTMSNI47-3), and AP019691 (pTMSNI47-4).
